# Potentials and current shortcomings in the cooperation between German centers for rare diseases and primary care physicians: results from the project TRANSLATE-NAMSE

**DOI:** 10.1186/s13023-021-02106-7

**Published:** 2021-11-24

**Authors:** D. Druschke, F. Krause, G. Müller, J. Scharfe, G. F. Hoffmann, J. Schmitt, TRANSLATE-NAMSE-Consortium

**Affiliations:** 1 Center for Evidence-Based Healthcare, University Hospital Carl Gustav Carus and Carl Gustav Carus Faculty of Medicine, Technische Universität Dresden, Dresden, Germany; 2 Center for Rare Diseases, University Hospital Heidelberg, Heidelberg, Germany; 3 Center for Child and Adolescent Medicine, University Hospital Heidelberg, Heidelberg, Germany

**Keywords:** TRANSLATE-NAMSE, General practitioners, Pediatricians, Centers for rare diseases, Interface management

## Abstract

**Background:**

The TRANSLATE-NAMSE project with the strengthening of the centers for rare diseases with their affiliation to the European Reference Networks was a major step towards the implementation of the German National Plan of Action for People with Rare Diseases establishing better care structures. As primary care physicians, general practitioners and pediatricians play a central role in the diagnosis of patients with rare disease, as it is usually them referring to specialists and rare disease centers. Therefore, the interface management between primary care physicians and the centers for rare diseases is of particular importance.

**Methods:**

In a mixed-method-approach an anonymous postal survey of 1,500 randomly selected primary care physicians in Germany was conducted with focus on (1) *knowledge* about a center for rare diseases and how it works, (2) in case of cooperation, *satisfaction* with the services provided by centers, and (3) *expectations and needs* they have with regard to the centers. In addition, in-depth telephone interviews were conducted with physicians who had already referred patients to a center.

**Results:**

In total, 248 physicians responded to the survey, and 15 primary care physicians were interviewed. We observed a wide lack of knowledge about the existence of (45.6% confirmed to know at least one center) about how to access rare disease centers (50.4% of those who know a center confirmed knowledge) and what the center specializes in. In case of cooperation the evaluation was mostly positive.

**Conclusion:**

To improve medical care, the interplay between primary care physicians and rare disease centers needs to be strengthened. (1) To improve the communication, the objectives and functioning of the rare disease centers should become more visible. (2) Other projects dealing with the analysis and improvement of interface management between centers and primary care physicians, as described in the National Plan of Action for People with Rare Diseases, need to be implemented immediately. (3) If the project is evaluated positively, the structures of TRANSLATE-NAMSE should be introduced nationwide into the German health care system to ensure comprehensive, quality-assured care for people with rare diseases with special consideration of the key role of primary care physicians—also taking into account the financial expenditures of this new care model.

## Background

### The German National Action for Rare Diseases (NAMSE) and TRANSLATE-NAMSE

Since the 1990s, rare diseases have become a priority in European policy, which has implications at the national level of the Member States. Efforts have been made to improve research, diagnosis, and care for people with rare diseases. Currently, we are in the situation that 25 EU member states have a national strategy on rare diseases under implementation [1].

The German National Action Plan for Rare Diseases (NAMSE Action Plan) has been established in 2013. The document includes policy suggestions and proposes actions in the fields of care/centers/networks, research, diagnostics and information management. Four years after the publication of the National Action Plan for Rare Diseases, more than half of the proposed measures have been implemented [2]. Nevertheless, many objectives are still underdeveloped, for example, the provision of reliable information on specialized care and research facilities to patients and care providers [3].

The core of NAMSE is the development of a three-partite center structure consisting of certified reference-, specialist-, and cooperation-centers (A, B, and C-centers), which are to work on an interdisciplinary basis. A-centers at university hospitals are contact points for patients with unclear diagnoses with the aim of coordinating further care. B-centers are specialized in certain diseases or disease groups. Outpatient care is provided in C-centers [4].

Now there are 31 centers for rare diseases in Germany. Parallel to the national developments, 24 European Reference Networks (ERN) were designed for linking centers of expertise across Europe to care of patients with rare diseases what require highly specialized treatments and concentrated expertise and resources [4]. One of the non-disease specific interdisciplinary tasks of the type A centers is to participate in the European reference networks for rare diseases [5]. Some of the disease-specific B-centers of the centers for rare diseases in Germany are networked within the ERN with partners throughout Europe [4].

The start of the TRANSLATE-NAMSE project (https://translate-namse.charite.de/) in 2017 was a major step towards the implementation of central measures of the German Action Plan and establishing better care structures for people with rare diseases. The Innovation Fund of the Joint Federal Committee (G-BA) supported over three years the cross-sectoral care model for people with rare diseases. The project consists of 12 partners (9 university hospitals and their centers for rare diseases, 2 health insurance funds, the patient umbrella organisation Achse e.V.) and 2 evaluating institutions. Some of the TRANSLATE-NAMSE rare disease centers are members of diagnosis-specific ERNs. The focus has been on providing both patients with unclear diagnoses and patients suspected of having a rare disease with newly developed IT-supported diagnostic procedures and innovative diagnostics, such as whole genome sequencing.

Unclear diagnosis means patients show unclear clinical pictures in which there is a high probability of a rare disease, but in which the present symptoms do not allow a clear diagnosis or main criteria of the diagnosis are not fulfilled or additional significant symptoms not typical for the diagnosis are present [5]. All conventional investigations that exclude existing suspected diagnoses should have been performed.

The criteria defined by the NAMSE Action Plan on how a center for rare diseases should be organized to ensure the best possible care for people with rare diseases were in the center of TRANSLATE-NAMSE. The extent to which patients are actually diagnosed more quickly and whether they are satisfied with the project processes is part of the evaluation and, in the event of a positive evaluation, the aim is for the structures to become part of standard care.

But only sufficient funding ensure that medical coordinators at the centers are available to answer questions from physicians and allows that the new care model of TRANSLATE-NAMSE will be sustainably integrated into the national system of standard care. So far it is known that there is still much to be done at the interface of physicians and centers to improve the care provision for people with rare diseases [6] the performance of the physicians is not sufficiently reflected in the German uniform Fee Scale for Medical Procedures (EBM, part of the Statutory health Insurance System) [7]. However, the Joint Federal Committee (Gemeinsamer Bundesausschuss, G-BA), the highest decision-making body of the joint self-administration in the German health care system, presented criteria in December 2019 that provide a basis for making the work of the centers for rare diseases visible and thus for ensuring that part of the additional costs of patient care can be financed.

### Evaluation of the cooperation between primary care physicians and centers for rare diseases

In order to create a comprehensive care structure for patients with rare diseases, networking with primary care providers and specialists outside the centers for rare diseases is essential. In Germany, primary care physicians occupy a key position in the care of patients with rare diseases. They are the first point of contact for low-threshold, patient-centered and holistic care [8]. As the first point of contact, the primary care physician accompanies and coordinates the treatment of patients and guides them within the framework of today's diverse treatment and therapy options.

If a rare disease is suspected, the best way to avoid diagnostic delays is to get clarification at a center for rare diseases [9]. Not only the cooperation of the various departments within a university hospital and the center for rare diseases play an important role in patient care, but also the primary care physicians and specialists in private practice. It is them who usually express the suspicion of a rare disease and forward the patient when they reach the limits of their expertise. Due to the rarity of the disease, often there is a lack of information available to health care providers. It is therefore important to find out to what extent general practitioners and pediatricians are familiar with the work of the rare disease centers and what expectations and obstacles exist for cooperation. Nine well-established centers for rare diseases at University Hospitals in Germany have participated in TRANSLATE-NAMSE. The question is how the quality and benefits of the inter-regional, multi-professional and cross-sectoral networks set up in the TRANSLATE-NAMSE project will be assessed by practitioners. In this context, it is important to understand the preferences, expectations and needs of general practitioners and pediatricians for the care of patients with rare diseases.

## Methods

The TRANSLATE-NAMSE project has investigated drivers and obstacles in the cooperation between the centers for rare diseases and primary care physicians. The expectations and needs in the care of patients with rare disease were evaluated by an anonymous postal survey of 1,500 general practitioners and pediatricians in Germany. The focus of the survey included (1) the knowledge of primary care physicians about a center for rare diseases and how it works, (2) in the case of cooperation, the satisfaction with the services provided by centers, and (3) expectations and needs with regard to the centers. In order to obtain a deeper insight and open feedback on the question complexes, additional telephone interviews were done with those primary care physicians who had already referred patients to a center for rare diseases within the TRANSLATE-NAMSE project.

### Quantitative methods: survey data

**Fig. 1:**
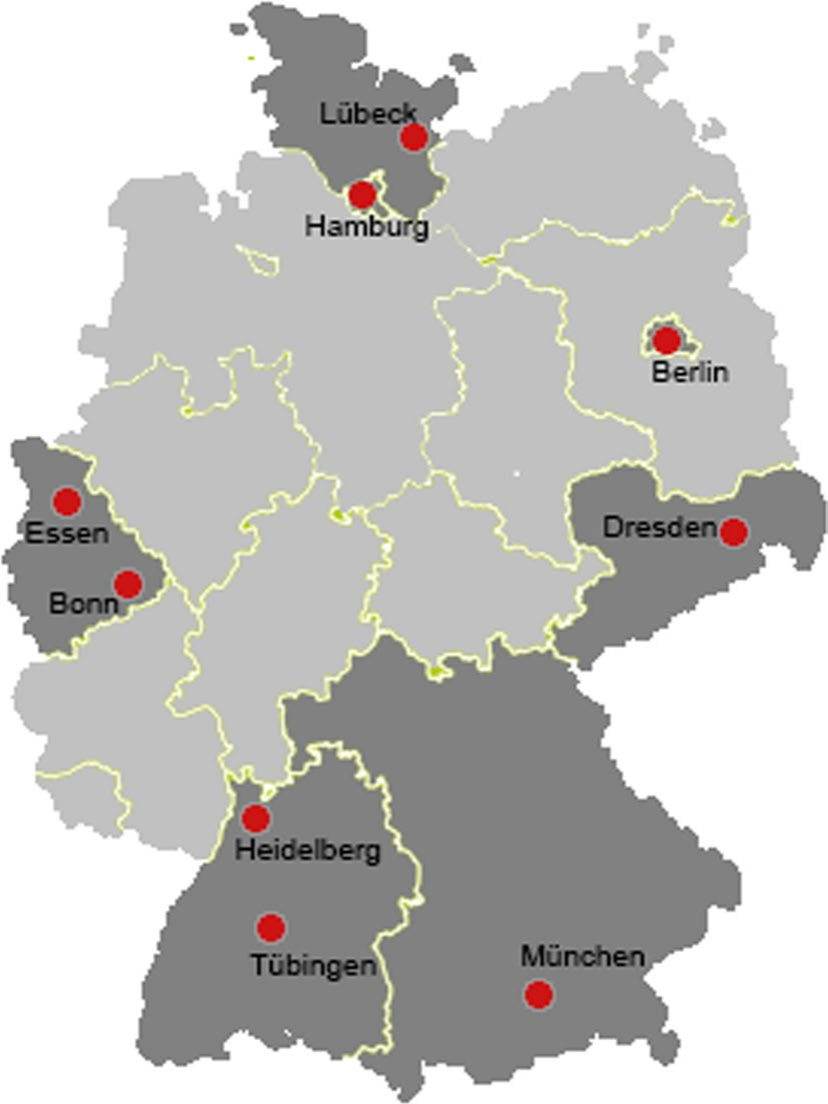
Participating centers in TRANSLATE-NAMSE in six regional areas (map was created using: https://www.mixmaps.de/deutschland/karte.html)

Survey data were collected with a two-page questionnaire of general practitioners and pediatricians. The survey focused on experiences with affected patients and diagnosing, used sources of information on rare diseases and the respective center for rare diseases, need assessment of support and if applicable the type and quality of cooperation for known centers. In questionnaire the physicians could select from a list which information sources on the topic of rare diseases they know. The contacted persons were selected as follows: The nine participating centers in TRANSLATE-NAMSE were grouped into six regional areas according to the divisions of the German Association of Statutory Health Insurance Physicians (“Kassenärztliche Vereinigung (KV)”): Berlin, North Rhine, Saxony, Hamburg and Schleswig–Holstein, Baden-Württemberg, Bavaria (see Fig. 1).

For address search the publicly accessible physician indices of the respective KV divisions were used. 250 addresses (170 addresses of general practitioners and 80 addresses of pediatricians) have been requested from each area and randomly selected as follows: As pediatricians play a specific role in the diagnosis of rare diseases [10], an oversampling of pediatricians of around one third were included in this weighted random sample.

### Qualitative methods: semi-structured telephone interviews

In addition to the survey information the qualitative method of semi-structured interviews was chosen to obtain in-depth information about relevant factors and barriers for the cooperation and communication with centers for rare diseases. Interviews were conducted with referring physicians at least one participating TRANSLATE NAMSE center (inclusion criteria). In day-to-day business, the respective center asked the referring physicians for their consent to the telephone-interview and for permission to pass on their contact details to the interviewer focused on the aim of conducting 18 interviews.

For the semi-structured interviews a theoretically sound and uniform interview guideline was developed with 15 open questions focusing on:

- the importance of rare diseases in everyday work
- experiences with affected patients or with patients suspected of having a rare disease
- used sources of information on rare diseases and the respective center for rare diseases
- need assessment of support from the physicians point of view and
- the type and quality of cooperation.

Guideline development and formulation of interview questions followed guidelines from the relevant methods literature [11].

For quality assurance purposes, the content of the semi-structured interview guideline was reviewed by various clinical experts from the TRANSLATE-NAMSE consortium. The interviews were conducted by a trained and experienced interviewer (DD) by telephone and audio-recordings. Each interview was transcribed verbatim. Based on these transcripts, a serial data analysis was performed using structured content analysis [12], which offers the possibility to link deductive and inductive development of categories [13] as recommended for the evaluation of guideline-based interviews [14].

For the analysis of the transcribed material, categories were formed consisting of both the direct transmission of the interview questions and the answers of the interviewees. The category system was tested and modified as part of the analysis of the individual interviews. All relevant text passages were extracted and put into their specific categories. During the process of examining and analyzing the individual interviews, the categorical system was modified and specified. Additional categories were developed when relevant and added inductively based on the collected text material up to the point the system became saturated (i.e., no new categories emerged [13]). In order to avoid a one-sided assignment, the entire process was carried out by two members (JuS, DD) of the working group [14]. For quality assurance purposes, the analysis process was discussed at regular intervals. All steps were processed with the qualitative data analysis (QDA) software MAXQDA version 2018.

### Data protection and data management

The participants in the survey received written information about the TRANSLATE-NAMSE project, the objectives of the survey and the voluntary nature of the participation. The questionnaire was returned anonymously, and the completed questionnaire was considered as consent to participate.

The participants of the interviews were informed in writing about the aims of TRANSLATE-NAMSE with a special focus on the protection of their data. The participants' consent was documented by signing the declaration of consent. With the participant's consent, the interviews were recorded for later transcription and all personal data was removed from the transcribed data set.

## Results

### Characteristics of the participants

A total of 248 general practitioners and pediatricians (47.6% female) responded to the survey (16.5% response rate; 15.8% for general practitioners and 18.1% for pediatricians). The majority (82.3%) worked in their own medical practice, 14.5% were hired in a medical practice or a medical care center (Medizinisches Versorgungszentrum, see Table 1).

**Table 1:** Characteristics of the survey-participants

	Frequencies (%)
Response rate	16.5
*Sex*	
Female	47.6
Male	52.0
No information	0.4
*Age group*	
30–39 years	8.1
40–49 years	21.0
50–59 years	44.8
60+	25.0
No information	1.2
*Medical specialty*	
General practitioners	64.9
Pediatricians	35.1
*Professional activity*	
Own medical practice	82.3
Hired in a medical practice or a medical care center	14.5
Hired in a medical care center and an university hospital	0.8
Hired in a medical practice and work in own medical practice	0.8
Hired in a medical care center and in a center for rare diseases	0.4
No information	0.4

Additionally, it was possible to conduct 15 in-depth interviews with physicians (7 female; 6 general practitioners, 6 pediatricians, 1 specialist in neurology, 1 specialist in orthopedics and 1 specialist in internal medicine) who have referred patients in a center for rare diseases. These 15 physicians were recruited by the TRANSLATE-NAMSE centers for rare diseases. Interview duration was on average 18 min (range 7–28 min).

### Survey results

The following table (see Table 2) summarizes the results of the survey questionnaire, which relate to general work of the physicians in care of patients with rare diseases. In the subsequent texts the integrated results of both methods (survey and interviews) are presented.

**Table 2:** Survey results

	Frequencies (%)
*Treating patients with or suspected of having rare diseases*	
Yes	82.7
No	17.3
*Number of patients with or suspected to have rare diseases per year*	
< 1	2.9
1–4	61.5
5–9	21.5
10+	8.8
No information	5.4
*Percentage of own patients referred to a center for rare diseases*	
0	44.4
< 50%	20.5
≥ 50 to < 100% years	7.3
100%	26.3
No information	1.5
*If patients with rare diseases have been treated, the diagnosis was made by whom? (multiple answers possible)*	
Own medical practice	28.3
Specialist colleagues	23.4
Hospital without center for rare diseases	31.2
Hospital with center for rare diseases	8.8
University hospital	41.5
Center for rare diseases	2.4
Don’t know	8.8
*Sources used for information on rare diseases (multiple answers possible)*	
Internet search engines	61.7
Textbook/journals	52.0
Special consultation hours at clinics	41.9
Personal contact with clinician	26.6
Orphanet (european information system for rare diseases)	15.3
Center for rare diseases	12.9
Self-help groups	5.2
Se-atlas (electronic platform of information)	1.2
Achse e.V. (umbrella organisation of self help groups)	0.8
Other	3.6
*Confidence in dealing with patients*	
Rather safe/very safe	12.1
Neither safe/unsafe	37.1
Rather unsafe/very unsafe	46.8
No information	4.0
*Desired support in care (multiple answers possible)*	
Contact person for specific rare diseases	73.8
Co-care of the patients	73.8
Support for diagnosis	64.5
Provision of general information	43.1
Information on clinical trials	15.7
None	1.2
Other	0.8
*Is the diagnostic process accelerated by center for rare diseases?*	
Probable/definitely	70.2
Partly	7.3
Definitely not/unlikely	5.2
Don't know	14.5
No information	2.8
*What is one's role in the care of patients with rare diseases? (multiple answers possible)*	
Co-carer	73.3
Coordinator	69.4
Stakeholder of the patients with rare diseases	31.0
Referring physician	27.4
None	0.4
Other	0.4

### Interview results

Because interview results are used to describe the survey questionnaire results in more detail, the presentation of the qualitative results follows the content structure of the questionnaire. Seven main categories were identified, each focusing on individual important aspects in the evaluation of the centers for rare diseases as seen by referring physicians. The respective main categories were (1) the quality of cooperation with the center for rare diseases, (2) information sources and flows, (3) structural framework in health care, (4) desired support from the center for rare diseases, (5) transition of patients, (6) characteristics of the respondents and (7) patients with (suspected) rare disease.

### Treatment of affected patients or patients with suspected rare disease in the doctor's office

In the survey, 82.7% of physicians confirmed that they were currently treating diagnosed patients or patients with suspected rare disease in their doctor's office or had treated such patients in the past. The statements in the in-depth interviews make it clear that primary care physicians often do not have standardized procedures for this type of patient: “The patient comes here, has her complaints, can't continue to study, and I don't know what to do.”

Overall, 9 of 15 interview participants described themselves as committed, persistent, and compassionate physicians who are concerned about clarifying the diagnosis in the interests of their patients: “I think patients have a right to know what they have, and if it is only psychological or psychosomatic, then it is also important to know because one treats quite differently. And this eternal journey from one doctor to another with the hope of finding something in the body and you don't find it, and then it just keeps the whole thing ongoing. That's why I'd rather say: “Okay, the sooner, the better,” rather than “beat around the bush” and then wait and see.” Or the interviewed mentioned “Yeah, he had visited me quite often. Of course, I also see, in comparison to other patients, his level of suffering and the symptom-complex he had. And, yes, I also felt the need to have a look and to find out what might be behind it, what the young man has.”

However, this commitment is not without additional resources: There are 2 of 15 interviewees who stress that they either do not have the resources or simply do not have sufficient knowledge of the disease in question to continue the specific care for the patients:

“The main responsibility for the whole thing, for organizing appointments and so on lies with the families. If they can't get it done for whatever reason, we get involved. But in terms of resources, we couldn't afford to coordinate it ourselves”.

### Expectations and needs of primary care physicians

All respondents indicated their expectations of working with a center with the multiple choice option of questionnaire. Most important for the primary care physicians was to have a contact person for certain rare diseases (73.8% of all mentions), to have ensured the patient's co-care (73.8%) and to receive support in diagnosis (64.5%). Access to clinical trials, which plays an important role in the field of rare diseases due to the lack of therapies, was rated less often as important. The fact that the centers are able to perform important tasks was also shown in the assessment of whether the diagnostic process is accelerated by centers for rare diseases. 70.2% of respondents stated that this was definitely or probably the case. Above all, the expertise provided by the centers seems to be decisive for the positive attitude of the interview participants: "…Yes, especially if it is rare, yes, then I rely on the competence of my colleagues, who are better acquainted with it through this specification or this professional specialization, also with rare ones, which I see once in a lifetime, they probably see once a year".

The question about support includes all the statements made by respondents regarding their requests for support services from the centers for rare diseases.

From the frequency of responses across different interviewees, conclusions can be drawn about their significance. Among the deductive categories (see Table 3) the most frequently mentioned by 6 respondents each are the desire for a contact person (preferably by the telephone) and the desire for support in making a diagnosis. Among the inductively formed categories under the category “Wish to centers for rare diseases,” the desire for feedback from the centers for rare diseases stands out (mentioned by 9 respondents), followed by the desire for a list or a brochure containing an overview of the centers for rare diseases as a whole, their specific focus, access modalities, and contact details (6 respondents).

**Table 3:** Desired support from the centers for rare diseases

What kind of support desires the physicians?	
Deductive categories (derived from the interview guide)	More information on rare diseases Telephone contacts Better availability by the telephone contact person Co-care Therapy recommendation Diagnostic support
Inductive categories	List with all center for rare diseases, their priorities and access modalities Center for rare diseases should inform about self-help groups The desire for more public relations from and for the center for rare diseases Feedback from center for rare diseases Better information for patients about their disease Low barriers to access to the center for rare diseases

The following table (see Table 4) summarizes the results of the survey questionnaire which relate to specific experiences with at least one center for rare diseases. In the subsequent texts the integrated results of both methods (survey and interviews) are presented.

**Table 4:** Survey results to specific experiences with at least one center for rare diseases

	Frequencies (%)
*Knowledge of at least one center for rare diseases*	
Yes	45.6
No	52.8
No information	1.6
*Used sources of information on this center for rare diseases (multiple answers possible)*	
Internet search engines	35.4
Medical network	34.5
Flyer/journals/media	19.5
Patients/relatives	14.2
Congresses	11.5
Medical association	4.4
Achse e.V. (umbrella organisation of self help groups)	0.9
Se-atlas (electronic platform of information)	0.9
Other	10.6
*Knowledge for access to a center for rare diseases*	
Yes	50.4
No	42.5
No information	7.1
*If access is known, how do you feel about the access*	
Very simple/simple	50.9
Neither nor difficult	22.8
Very difficult	22.8
No information	3.5
*In case of cooperation: form of cooperation with the center*	
Co-caring	42.5
Support for diagnosis	36.3
Information about rare diseases	18.6
Involvement in research projects	4.4
None	27.4
Other	1.8
*In case of cooperation: evaluation of communication*	
Very good/good	59.1
Average	15.2
Poor/very poor	16.7
No information	9.1
*In case of cooperation: is cooperation helpful*	
Very helpful/rather helpful	75.8
Partly	12.1
Rather not helpful/not helpful	4.5
No information	7.6
*In case of cooperation: satisfaction with cooperation*	
Very satisfied/rather satisfied	80.3
Partly	4.5
Rather not satisfied/not satisfied	9.1
No information	6.1

### Knowledge about a center for rare diseases

In the survey, 45.6% of physicians stated that they knew a center for rare diseases, 52.8% denied this. The majority reported having heard about a center via the internet (35.4%) or their own medical network (34.5%). Self-help organizations such as Achse e.V. and the online se-atlas, which lists all centers in Germany, did not count as reference points with only one mention each.

The interviews revealed a more differentiated picture: For instance, university education, work at the university hospital and publications have also been cited as sources of knowledge. It has been repeatedly remarked that, despite the knowledge of a center, there is still uncertainty as to how it works: "… what center xy has specialized in is beyond my knowledge …" or "… So, when it says 'rare diseases', it wasn't clear to me that they were so different, (…) But why, yes? Does everyone work on rare diseases, what is the difference? Or why don't they work together on that?” This obviously also leads to physicians not being sure when they can refer a patient to a center: "And that there is simply a consultation hour or a telephone number where you can easily register the difficult patient where you can't get any further."

### Communication process with a center for rare diseases

In the survey, the respondents, who know at least one center for rare diseases assessed how they felt about communicating with the center. 59.1% of statements on perceived communication classify them as good to very good, 15.2% as average and 16.7% as poor or very poor. In the interviews (Table 5), it became clear that the communication problems are mainly the fact that physicians do not know after what period of time they will receive feedback and whether their patients have already received an appointment at all: "…as I was with the senior physician, I already had, I say, telephone contact in advance. And then there was a letter where there was a little bit in it. And there was then, I say, then it became quiet" In addition, the physicians receive the reports of the centers either delayed or not at all according to their own statements: "So from xy I have no report. Well, I only got that from the mother…that's of course stupid when I refer to the center for rare diseases … that I won't get a report any more." or "… Normally we also get a report sent to us. Well, it doesn't always work…".

**Table 5:** Evaluation of the quality of cooperation and communication from the point of view of the interviewed physicians

Evaluation of the quality of cooperation and communication from the point of view of the interviewed physicians		
	cooperation	communication
Negative	There is no cooperation as there is no access Physician put the diagnosis in question Waiting time until feedback for the report of findings	No transparency about processes and results in the center of rare diseases No feedback Unclear communication—> physician does not know who or how is being communicated
Positive	Therapy recommendation is useful Friendly, professional cooperation Rapid diagnostic communication Positive effect of the centers on diagnostics Better health care provision by the center of rare diseases	Short-term accessibility Fast feedback Regular findings reports Information telephone available around the clock

The evaluation of communication with the centers for rare diseases correlated with satisfaction (p < 0.01). If they rated communication with the centers as good or very good all respondents are 100% satisfied with the centers for rare diseases. 58.8% were rather or very dissatisfied with the centers for rare diseases if they evaluate the communication as poor or very poor.

### Access modalities

Of the 50.4% of respondents who reported knowledge on the steps to get access to at least one center for rare disease, access was evaluated (Table 6).

**Table 6:** Evaluation of the access modalities to centers for rare diseases

Evaluation of the access modalities to centers for rare diseases from the point of view of the interviewees physicians	
Negative	Access unknown Complex access arrangements Unreasonable laboratory requirements beforehand Waiting time until feedback for the access processing Waiting time until appointment allocation Referred to centers for rare diseases in the region
Positive	Fast and easy access Short waiting times until feedback Short waiting times until the appointment allocation Routinely established allocation to certain centers for rare diseases

The variable satisfaction with centers for rare diseases and access to them are closely related (*p* < 0.01). For example, 95.8% of respondents are quite or very satisfied if access to the centers is considered to be simply or very easily. If access to the centers for rare diseases was considered difficult or perceived as very difficult, satisfaction with the centers for rare diseases also decreases. For example, the majority of respondents who found access to the centers for rare diseases difficult or very difficult were dissatisfied (64.3%).

### Benefit

To the question of whether they considered the center's performance to be helpful and how satisfied they have been with it, 75.8% of all mentions rated helpful, and 80.3% of all mentions rated to be satisfied with the performance. Only 4.5% considered the center's services as unhelpful and 9.1% were dissatisfied. While the critical voices rather see that it is not always possible to make a diagnosis "…Yes, whether that was the bottom line in the end, I dare to doubt….Yes everything has been checked out, but the bottom line is that no change in the general condition of the patient is achieved as a result…", others perceive the support of the center in the diagnosis as an enrichment: "Well, of course this is very helpful. Because, of course, you do it in cases where you yourself have the feeling: 'Oh, I can't get any further there'. And I have to say that we are of course always very grateful. And that usually works well, too."

### Strucural framework conditions

The interviewees also addressed the structural framework conditions (see Table 7) in the care of rare disease patients.

**Table 7:** Structural framework conditions

Structural framework conditions in the health care system	
Systemic problems mentioned by the physicians	A problem of the spatial distance to the center for rare diseases Urban/rural differences in the health care provision possibilities Scarcity of resources of physicians Pressure from the health insurance companies Waiting time for outpatient (further) therapy Problem of alternative healing methods Criticism of the politically desired “24/7 all-round health care provision”

Also the strict separation of diagnostic procedures by sector (outpatient/inpatient) and the barriers for the use of new communication forms (e.g. video chat) and the obstacles to the implementation of digital patient records (data protection or lack of knowledge of physicians) were criticized.

In this context, respondents commented on the problems they perceived as inherent to the health care system in the health care provision for patients with rare diseases. The problem of the spatial distance between the physician or the patient and the respective center for rare diseases was mentioned: “Whereby I can, of course, already see that we simply have this advantage, that we know our colleagues personally, that we are close by, that is no longer natural for someone who is now somewhere more in the countryside, where perhaps the next university is 60 km or 100 km away, it is, of course, probably more difficult.” or “These are not standard examinations that one can somehow do in our normal laboratory. And until we found out how, when, and which urine sample I collected and sent to what center for examination, it would be absolutely beyond our resources and the scope of this discussion.”

Thematically close is the difference in the structure and the offer of health care provision between city and countryside. The scarcity of resources for the physicians was also brought up. Other problem areas addressed have been the long waiting times until outpatient (follow-up) therapies are started, the alternative treatment methods perceived as problematic („Also, he came up with several ideas of his own that were really abstruse and where I saw the risk of being taken out money of his pocket. Of course, I had to protect him from taking such paths, with some kind of electromagnetic method or whatever.“), and the perception of a politically desired “24/7 all-round health care provision” for patients, which was cited as the reason for the long waiting times at the centers.

Another important factor in the structural framework conditions for the health care provision is the availability of self-help groups. Here, too, the respondents noted differences in health care provision possibilities between city and countryside.

Seven of the interviewed physicians in private practice stated that they provide information about self-help groups, while four stated that they do not provide information about self-help groups. Two interviewees stated that they did not select a specific self-help group for their patients. One physician explicitly rejects self-help groups; two physicians doubt the meaningfulness of self-help groups for their respective patients and two physicians state that their patients or their relatives would seek self-help groups themselves.

## Discussion

This large nationwide investigation on the cooperation between primary care physicians and centers for rare diseases used a mixed methods approach and thus combined the advantages of representativeness from the survey with the ability to get individual in depth insights from the interviews. This study has several key findings that are very important not only for the German healthcare system, but also for the provision of care for people with RD in all countries. However, when interpreting the results of the survey, it must be taken into account that the response rate was low (16.5%) and that most of the respondents are physicians who have already treated patients with rare diseases. By querying the primary care physicians it became clear that not all primary care physicians have an exact definition of rare diseases ad hoc. The data underline that rare diseases are a very real problem for a large number of primary care physicians. For example, 86% of respondents to a survey [15] and about 75% of respondents to an online survey [16] reported that they had already treated patients with suspected or confirmed rare diseases. These figures affirm once again the relevance of rare diseases and the support needed for primary care of people and relatives in this area. It became clear that respondents information tools specially designed for the field, such as the se-atlas, orphanet or patient self-employment organisations such as ACHSE e.V. or EURORDIS are mostly unknown.

The centers for rare diseases are rarely regarded as a source of information on the topic of rare diseases. Especially with regard to their intended function as a research institution for rare diseases, the values obtained here are of concern, as they suggest that that the expertise gathered here in the field of rare diseases does not reach potential referrers.

A remarkable result is the self-assessment of just under half (46.8%) of physicians who rate themselves as rather or very unsafe in dealing with patients with rare diseases. Only 12.1% of participants assessed their treatment of rare diseases patients safe or very safe. One reason for this uncertainty may be in the absence of knowledge of rare diseases itself and also with regard to sources of information on the topic.

The expressed requests for support recapture the shortcomings in the field of care for patients with rare diseases: there is a lack of information on rare diseases, lack of information regarding sources of information in the field and they feel not sufficiently supported in the treatment of patients with (suspicion of) a rare disease, but also have not sufficient knowledge of support in their region. Neither the region nor the affiliation with general practitioners or pediatricians leads to differences in the quantitative survey assessment of cooperation with the Centers for rare diseases.

Attitudes towards self-help groups tend to be mixed, it is noticeable that ACHSE e.V. as the largest patient self-help organisation for rare disease patients and their families is mostly unknown to the interviewees. The quantitative analysis of the questionnaire data showed that the diagnosis of a rare disease was largely carried out in university hospitals (41.5%). About a quarter of the rare diseases have been identified in the respective general practitioner or pediatric practices and just under 3% of the diagnoses were made by a center for rare diseases impact (see Table 2). At first glance, this result seems at least striking, but it can be explained by the lack of knowledge about centers for rare diseases and the access to them. Only 45.6% of general practitioners and pediatricians know about a center for rare diseases. This result is astonishing and reveals a significant information deficit on the part of primary suppliers. In view of the fact that since 2009 more than 30 centers for rare diseases have been located throughout Germany [17], these values are in terms of the level of knowledge of centers for rare diseases downright frightening. It is clear that the problem of rare diseases and the supply of centers in the region are not yet known. Especially because of the large number of heterogeneous patients who are treated in practices with suspected rare disease, the reported lack of information has to be eliminated. This should be implemented in view of the sources of information used by doctors, in particular through information advertising on the internet and in specialist journals.

The qualitative analysis of the results shows that primary care physicians want to have an overview of the various centers for rare diseases and their respective priorities as well as their respective access modalities. In addition, in the course of the evaluation of the telephone interviews, it became clear that a large part of the communication does not take place between the centers for rare diseases and directly with the physicians treating the patients, but that the patient plays an important role in the mediation of appointments, reports and treatment options.

The care of patients with rare diseases places high demands on the treating physicians. Diagnosis is often only made after many years. It is therefore important to take time to share information about the diagnosis, the associated options for action and the associated risks with patients. However, the relevant information is missing. For additional exchange and research, information on certain information platforms such as se-atlas, Orpha.net or patient self-help organizations such as ACHSE e.V. or EURORDIS is of great importance.

The study provides a comprehensive picture of the evaluation of the centers for rare diseases by the referrers. On the one hand, the quality of cooperation is clearly positive in the quantitative survey. On the other hand, there is also clear criticism regarding the long waiting times, access to the individual centers and the communication between the ZSE and the physicians.

The following can be summarized for the study presented here: In principle, there is a quite impressing satisfaction among physicians who refer their patients to a center for rare diseases with their work. However, many general practitioners do not know that centers for rare diseases exist, so it can be assumed that in Germany there are often unnecessary delays in diagnosis due to expertise being obtained too late or not at all. Even if general practitioners have the knowledge of a center, one of their criticisms is that it is often unclear what the access modalities are. The lack of information and uncertainty may increase the likelihood that the GP or pediatrician will hesitate to refer a patient to a center which again results in delay of diagnosis.

The Centers for rare diseases are rarely used in their function as a source of information on rare diseases, their access modalities are generally relatively unknown. In this area, there is a clear need to increase awareness about the centers, the characteristics of their work and access modalities. The possibility of a telephone contact person would also be possible for rural areas.

These results are important because they allow timely steps to be taken to improve work at the interface between centers and referring physicians and to strengthen existing structures that have already proved useful. For instance, in order to get an idea of the health economic effects of a delayed referral of patients to a center for rare diseases, the TRANSLATE-NAMSE project also carries out a health economic evaluation.

### Strenghts and limitations

The mixed methods approach combines the advantages of representativeness from the survey with the ability to get individual in depth insights into the cooperation between general practitioners, pediatricians and centers for rare diseases from the interviews. The mixed methods-study showed reveals a lack of knowledge in a theoretical (e.g. the existance of centers for rare diseases) and practical manner (e.g. access to a center) for physicians. In cases of cooperation with processes of centers for rare diseases, which are rarely transparent for referrers. On the other hand the cooperation was evaluated as expedient and supportive e.g. for diagnosing rare diseases. With regard to ensuring adequate care for patients with rare diseases, this result is highly valuable. The results on relevant cooperation characteristics provided important information for future improvements overcoming lack on information and transparency.

In addition to its strengths, our study also has limitations. The survey response rate was low and the respondents have already followed patients with rare diseases. This is associated with a selection bias. In the qualitative part, the interviewees are a pre-selected group, as at least one contact with a center for rare disease was a prerequisite for inclusion. In addition the interviewees have a specific interest in scientific research on rare diseases and may be engaged above-average. This commitment is also expected in treatment of patients with (the suspicion of) rare diseases and is probably reflected in the self-perception of the physicians interviewed. Furthermore, recall bias may distort the quantitative results. The direct interview contact with the referring physician after assigning a patient to a TRANSLATE-NAMSE center prevented recall bias in describing the character of cooperation with a center. For the quantitative study, the random sample was used to counteract the selection bias.

## Conclusions

In order to improve the quality and efficiency of care in the field of rare diseases in the long term, greater efforts must be made to raise awareness of both the centers for rare diseases, their respective access routes and sources of information on rare diseases. It is also essential for the centers for rare diseases to improve networking between medical service providers and communication with general practitioners.

As a result of the study, the following suggestions for improving communication between physicians and centers can be proposed:

(1) The functioning and objectives of the Centers for Rare Diseases must become more visible. To this end, existing homepages should be made more user-friendly, i.e. the access modalities should be clearly explained. In addition, it might be useful to provide feedback to the physicians about the expected duration of their patients' treatment and when at the earliest a feedback or diagnosis can be expected. A reference to the so far not sufficiently known se-atlas should be mandatory for all websites of centers for rare diseases, since physicians can also find out independently who they can turn to. More information on rare diseases must be provided in the form of specialist congresses for rare diseases themselves, but also within the various specialist disciplines and through publications in various journals [18].

(2) Other projects dealing with the analysis and improvement of interface management between centers and physicians, as described in the NAMSE Action Plan, need to be launched. The results should not only be disseminated and discussed throughout Germany, but also at the European and international level in order to make the findings available for supranational structural improvements in the care of people with rare diseases. The existing structures on national and European level, such as se-atlas, orpha.net, Achse e.V or EURORDIS need to become more visible to care providers and patients.

(3) If the project is evaluated positively, the structures of TRANSLATE-NAMSE should be introduced nationwide into the German health care system to ensure comprehensive, quality-assured care for people with rare diseases with special consideration of the key role of primary care physicians—also taking into account the financial expenditures of this new care model. The final report and the evaluation report of TRANSLATE-NAMSE are currently being evaluated. So far it is known that there is still much to be done at the interface of physicians and centers to improve the care provision for people with rare diseases [6] and that the performance of the physicians is not sufficiently reflected in the German uniform Fee Scale for Medical Procedures (EBM) [7]. The Joint Federal Committee (Gemeinsamer Bundesausschuss, G-BA), presented criteria that provide a basis for making the work of the centers for rare diseases visible.

## Data Availability

The data that support the findings of this study are available from corresponding author but restrictions apply to the availability of these data, which were used under license for the current study, and so are not publicly available. Data are however available from the authors upon reasonable request and with permission of Innovation Fund of the Joint Federal Committee.
